# Functional and Structural Succession of Soil Microbial Communities below Decomposing Human Cadavers

**DOI:** 10.1371/journal.pone.0130201

**Published:** 2015-06-12

**Authors:** Kelly L. Cobaugh, Sean M. Schaeffer, Jennifer M. DeBruyn

**Affiliations:** Department of Biosystems Engineering & Soil Science, University of Tennessee, Knoxville, Tennessee, United States of America; Graz University of Technology (TU Graz), AUSTRIA

## Abstract

The ecological succession of microbes during cadaver decomposition has garnered interest in both basic and applied research contexts (e.g. community assembly and dynamics; forensic indicator of time since death). Yet current understanding of microbial ecology during decomposition is almost entirely based on plant litter. We know very little about microbes recycling carcass-derived organic matter despite the unique decomposition processes. Our objective was to quantify the taxonomic and functional succession of microbial populations in soils below decomposing cadavers, testing the hypotheses that a) periods of increased activity during decomposition are associated with particular taxa; and b) human-associated taxa are introduced to soils, but do not persist outside their host. We collected soils from beneath four cadavers throughout decomposition, and analyzed soil chemistry, microbial activity and bacterial community structure. As expected, decomposition resulted in pulses of soil C and nutrients (particularly ammonia) and stimulated microbial activity. There was no change in total bacterial abundances, however we observed distinct changes in both function and community composition. During active decay (7 - 12 days postmortem), respiration and biomass production rates were high: the community was dominated by Proteobacteria (increased from 15.0 to 26.1% relative abundance) and Firmicutes (increased from 1.0 to 29.0%), with reduced Acidobacteria abundances (decreased from 30.4 to 9.8%). Once decay rates slowed (10 - 23 d postmortem), respiration was elevated, but biomass production rates dropped dramatically; this community with low growth efficiency was dominated by Firmicutes (increased to 50.9%) and other anaerobic taxa. Human-associated bacteria, including the obligately anaerobic *Bacteroides*, were detected at high concentrations in soil throughout decomposition, up to 198 d postmortem. Our results revealed the pattern of functional and compositional succession in soil microbial communities during decomposition of human-derived organic matter, provided insight into decomposition processes, and identified putative predictor populations for time since death estimation.

## Introduction

Decomposition of organic matter is an integral process in all ecosystems. Most research on terrestrial ecosystems focuses on decomposing plant litter due to its sheer dominance as an input [1]. Less well studied is the decomposition of animal carcasses, which are a chemically and physically distinct organic residue input. Due to a narrow carbon to nitrogen ratio (5:1 to 8:1), high water content, and a wide diversity of labile nutrients, carcasses decay much faster than plant litter [2]. Carcass decomposition releases a diversity of compounds into the soil, including adipose tissues, volatile fatty acids (primarily butyric and propionic acids), organic acids (e.g. acetic and oxalic acids), organic nitrogen (nucleic acids, peptides, amino acids), and phenolics [3]. This localized, ephemeral decomposition event results in a “hot spot” and “hot moment” of enhanced biogeochemical cycling in the soils below (vis a vis [4]). Indeed, previous studies have noted increases in microbial respiration rates, nitrogen mineralization and biomass carbon underneath carcasses [5–8]. Scientists have adopted a fertility island concept to describe the localized ecosystem area created by a decaying carcass, referred to as a ‘cadaver (or carcass) decomposition island (CDI)’ [9]. These hot spots, or CDIs, contribute to landscape heterogeneity, biodiversity, and terrestrial biogeochemical cycling [2,8–11]. Understanding carcass or cadaver decomposition processes may also have direct applications for forensic science [9].

It is well accepted that microbes are responsible for recycling carcass-derived organic matter, yet we know surprisingly little about the microbial communities responsible. Decomposition of complex organic matter is carried out by multiple microbial taxa and there is emerging evidence that community composition affects decomposition rates and processes [12–14]. Plant litter studies have identified soil bacteria and fungi associated with decomposition [15,16], but soil taxa associated with carcass decomposition have not been thoroughly examined. In addition to the soil decomposers, carcasses carry a dense commensal microbial community (microbiome) which is presumably introduced to the soils as decomposition progresses. We know that commensal microbes mediate the initial tissue decomposition in the days following death [17,18], but it is unknown if they persist or contribute to decomposition beyond this initial stage.

As a carcass decomposes, there is a documented succession in insects, invertebrates and other fauna [2,19,20]. These successional patterns are used to estimate post mortem interval (PMI), or time since death, which can be key evidence in forensic investigations. Given the limitations of current PMI estimation methods, the succession of microbes and their utility in estimating PMI has garnered great interest in the forensic community [17,21–27]. There is evidence that microbes, too, follow regular functional and perhaps taxonomic patterns in these highly dynamic CDIs: with respect to carbon utilization, increases in soil respiration and microbial biomass during active decay are typical [6,28,29]. With respect to nitrogen cycling, enhanced protease activity [5] and free amino acid turnover rates [8] have been documented, along with increased nitrogen species (peptides, amino acids, ammonium, and ninhydrin-reactive nitrogen) [6,8,30]. Other functional shifts in CDIs include increased phosphodiesterase and lipolytic activity [5,31]. From plant litter studies, we know that the functional changes in decomposer communities are often mirrored by compositional changes [15,32–34]. Compositional shifts in microbial communities on (or in) cadavers or carcasses have been documented [17,23–26]. Fewer studies have examined soil microbial communities, but these also suggest that soil communities exposed to decomposition products are compositionally altered [22,23,35].

The aim of this study was to investigate successional dynamics of microbial communities involved in decomposition of human-derived organic matter in a terrestrial ecosystem. We hypothesized that the microbial communities in CDI soils undergo successional shifts in both function (biogeochemical cycling and activity) and structure (community composition and relative abundance of taxa). Specifically we hypothesized that periods of increased microbial activity during decomposition was associated with changes in community composition. We additionally hypothesized that members of the human microbiome would be introduced to the soil environment, contributing to the altered structure, but would not persist outside their natural environment. To answer our questions, we studied replicate human cadavers decomposing in a natural terrestrial environment at the University of Tennessee Anthropology Research Facility (ARF), a unique outdoor human decomposition laboratory.

## Materials and Methods

### Study site and sample collection

The study was conducted at The University of Tennessee Anthropology Research Facility (ARF) in Knoxville, Tennessee (35°56' 28" N, 83°56' 25" W), a 1.3 acre outdoor laboratory dedicated to the study of human decomposition. The site is a preserved temperate deciduous forest with a well-drained fine textured clayey soil [36]. The annual mean high and low temperatures were 21.3 and 10.9°C, respectively, with a mean relative humidity of 71.3% and a mean yearly rainfall of 121.5 cm (data collected from the University of Tennessee Gardens Meteorological Station, located 0.5 km from the ARF).

In total, four cadavers (two white males and two white females) were used in this study, which were donations to the University of Tennessee Forensic Anthropology Center for the W. M. Bass Donated Skeletal Collection (http://web.utk.edu/~fac/collection.html). As no living human subjects were involved, this work was exempt from review by the University of Tennessee Institutional Review Board. No preference was employed for sex, age, ancestry, weight, etc. The University of Tennessee protocol for accepting donations ensured the individuals did not have communicable diseases. The bodies were not autopsied or embalmed; they were immediately refrigerated after death and placed at the ARF within three days. Their age at time of death ranged from 60 to 90 years and weights from 56 to 77 kg, and all died of natural causes. The four cadavers (referred to as A3, B4, C5, D6) decomposed through the summer and fall of 2012 when the average air temperatures were 22.4, 27.8, 26.0, and 17.2°C, respectively. We also elected to examine gut samples from each cadaver as a reference, since the gut harbors a highly dense microbial community which is compositionally distinct from soils and other environments [37]. Immediately before placement, the cadaver’s gastrointestinal tract was sampled: the caecum was swabbed via a small incision, which was re-sealed with standard duct tape as previously described [26]. The caecum swabs were transported on ice and stored at -20°C until DNA extraction. Before the cadaver was laid on the soil, an initial soil sample was collected from the placement site. Using the methodology detailed in Parkinson et al. [22], an open weave mesh was placed underneath prone cadavers to allow for minimal disturbance while rolling aside for sampling of the soil underneath. Although this facility has been continuously used since 1980, every effort was made to obtain plots for the study that, to our knowledge, had not been previously used for decomposition studies.

Soil sampling started before cadavers were placed on the soil (“Initial”), and continued until cadavers reached a late advanced decay stage (S1 Table). The frequency of sampling was determined by the approximate stage of decomposition (Fresh, Bloat, Active Decay, Advanced Decay, and Late Advanced Decay, determined after Payne [38]), with attempts to capture each stage at least once. Therefore samples were taken more frequently during the first few weeks, as this was when decomposition proceeds most rapidly, then less frequently once cadavers reached Advanced Decay and changes were occurring more gradually. The final samples (“Advanced III”) were taken at 87, 198, 83 and 114 days for cadavers A3, B4, C5, and D6, respectively. At each sampling time, the top 0–3 cm of soil beneath the torso was sampled using a 0.8 cm corer; approximately 20 cores were randomly collected and composited. In addition, at each time, a soil sample was collected in the same manner from a control site located approximately 2 m away from each cadaver which had not been exposed to decomposition. All composite samples were sieved using standard soil sieve No. 10 (2 mm mesh) to remove plant roots, rocks, insects, hair etc. A fraction was stored at -20°C until DNA extraction, while the rest was used immediately for soil analyses.

### Soil analyses

Soil was extracted with 2 M KCl (1:5) for 1 h. The filtered extract was used to measure total extractable carbon via combustion/catalytic oxidation and total extractable N via combustion/chemiluminescence detection (Shimadzu TOC/TN-VCPH). Nitrate, ammonia and phosphate were measured via colorimetry (Skalar autoanalyzer). pH was measured in distilled water (1:2). Microbial biomass production rates were measured using a ^3^H-leucine incorporation method adapted for soil bacteria [39]. Microbial respiration was measured using a sodium hydroxide carbon dioxide trap and titration [40]. Gravimetric water content was determined to standardize the microbial activity and soil chemistry data by the dry weight of soil.

### DNA extraction and sequencing

Bacterial community structure analysis was done for select soil samples, based on decomposition stage. Since cadavers were placed at different times, there were slight differences in temperature and climate which resulted in different rates of decomposition. While carcass decomposition on the soil surface is not a process with distinct stages, categorization of common phenomena is a typical approach in forensic taphonomy. Daily photographic images of the cadavers were reviewed to categorize each cadaver by stage, using the five stages identified by Payne [38], plus intermediate stages to provide a higher resolution: Initial (before placement of cadaver), Bloat, Bloat-Active, Active, Active-Advanced Decay, Advanced Decay I, Advanced Decay II, Advanced Decay III (S1 Table). Community analysis was done on a soil sample from each of the eight stages for each cadaver plus eight non-cadaver control samples and the gut swab for each of the four cadavers (44 unique libraries) (S1 Table). DNA was extracted using the PowerLyzer PowerSoil DNA Isolation Kit (MOBIO Laboratories, Inc. Carlsbad, CA.) and PowerLyzer instrument (MOBIO), with an added heated incubation step to increase yield: samples were incubated at 65°C for 10 minutes in a water bath and at 95°C in a heat block for another 10 minutes prior to bead-beating. The remaining protocol was unchanged.

DNA was sent to the Genomic Services Lab at Hudson Alpha Institute for Biotechnology (Huntsville, AL) for library preparation using universal bacterial primers 515F and 806R [41] to amplify the V4 region of bacterial 16S rDNA, and sequencing on the Illumina MiSeq platform to generate 150 bp paired-end reads. DNA extractions from four randomly selected samples were sequenced in duplicate to assess technical reproducibility. Reads were processed with Mothur (v.1.33.3), following the MiSeq SOP [42] (S1 Text). Briefly, sequences with homopolymers longer than 8 nucleotides or containing ambiguous bases were removed. Remaining sequences were aligned to a SILVA bacterial reference library v102 [43] and trimmed to 291 bases that started and ended at the same alignment position. Reads were binned into operational taxonomic units (OTUs) according to their taxonomic classification at the genus level. The reads were subjected to the UCHIME chimera removal algorithm and the SILVA reference alignment was used to generate best species classification. After screening, 11,174,851 reads remained, available in MG-RAST (accession numbers 4629149.3–4629196.3). For diversity analysis, the number of sequences in each sample was normalized by randomly subsampling the number of sequences present in the smallest sample (121,340 reads) to eliminate the effect of uneven sampling depth. One sample, an Active Decay sample from cadaver C5, was removed from our analysis due to inadequate coverage: it contained only 5.7% of the average number of sample reads. The Simpson Diversity index and Chao richness were calculated for the subsampled libraries in Mothur.

### Enumeration of bacteria via quantitative PCR

To enumerate copies of bacterial 16S rRNA genes, qPCR with universal bacterial primers 1055F and 1392R were used as previously described [44]. Each 25 μl qPCR reaction consisted of 12.5 μl Maxima SYBR Green/Fluorescein qPCR Master Mix (2X) (Thermo Fisher Scientific Inc., Waltham, MA), 5.5 μl of nuclease-free water (Thermo Fisher Scientific Inc., Waltham, MA), 1 μl of 1055F primer (10 μM), 1 μl of 1392R primer (10 μM) (Eurofins MWG Operon, Huntsville, AL) and 5 μl template DNA diluted 1:100 in nuclease-free water. Standards consisted of a bacterial plasmid containing a cloned 16S rRNA gene: the 16S rRNA gene was PCR amplified from genomic DNA of a *Sphingomonas* isolate using the universal bacterial primers 8F and 1492R and cloned with the pGEM-T Vector System (Promega Corporation, Madison, WI) according to manufacturer’s directions. Plasmids were extracted using the Wizard Plus SV Minipreps DNA Purification System (Promega) and diluted to create standards from 10^8^ to 10^4^ copies per reaction. qPCR reactions were performed in triplicate, alongside template-free controls, on a C1000 Thermal Cycler with a CFX96 Real Time System (Bio-Rad, Hercules, CA) using the following protocol: 95°C for 10 min, then 40 cycles of denaturing at 95°C for 30 s, annealing at 51°C for 25 s, and extending at 72°C for 25 s. Gene copy numbers were determined using the regression equation relating threshold cycle (C_T_) to copies in the standards. Standard curves had a R^2^ > 0.969 in all cases with an average efficiency of 100.6% (data not shown).

To identify and track the fate of human-associated microbes in the soil, we enumerated a representative bacterial population from the human gut microbiome: following the method of Layton et al. [45], human-associated *Bacteroides* were enumerated throughout the decomposition process and at three time points after the cadaver remains were removed. Primers HuBac566f and HuBac692r were designed from partial alignments of *Bacteroides* 16S rRNA genes from fecal source libraries and GenBank. Each 25 μl qPCR reaction consisted of 12.5 μl ABsolute Blue qPCR Master Mix (Thermo Fisher Scientific Inc., Waltham, MA), 6.5 μl nuclease-free water (Thermo Fisher Scientific Inc., Waltham, MA), 1.5 μl of HuBac566f primer (10 μM), 1.5 μl of HuBac69r primer (10 μM), 0.5 μl HuBac594Bhqf probe (10μM) (synthesized by Eurofins MWG Operon, Huntsville, AL) and 2.5μl template DNA (diluted in nuclease-free water to 1:10 for soil, 1:100 for gut samples). All qPCR reactions were performed in triplicate. The plasmid used for the standard curve was obtained from the A. Layton lab group (UTK) and is described in Layton et al. [45]. The standard curve ranged from 2.5 x 10^2^ to 2.5 x 10^7^ plasmid DNA copy number per reaction. Standard curves had a R^2^ > 0.989 in all cases with an average efficiency of 84% (data not shown).

### Statistical analysis

Since the cadavers decomposed at different temperatures, the rates of decomposition varied. In order to combine the four cadavers as experimental replicates, data from each cadaver were binned by decomposition stage. Then, data from each stage for replicate cadavers were combined and subjected to graphical and statistical analysis. For the microbial activity data, a ranked mixed model ANOVA (SAS software) was used to analyze the statistical differences between stages, treatment (CDI and its respective control) and their interaction. Tukey-Kramer’s multiple comparisons tests were run and differences with a p-value less than 0.05 were regarded as statistically significant. To reveal significantly changing OTUs across stages, STAMP software used ANOVA with a Tukey-Kramer Post-Hoc test (p < 0.05)[46]. Unclassified reads were used only for calculation frequency profiles. Statistically significant OTUs were filtered with an effect size (η^2^) of 0.50 or larger and contained at least one stage in which the population was higher than 0.001%. Non-metric multidimensional scaling (NMDS) was used to visualize the phylogenetic distance (Bray-Curtis similarity) between the bacterial communities at each stage for the four cadavers. The taxonomic abundance data from Mothur was imported into the Plymouth Routines In Multivariate Ecological Research v6 (Primer v6, Lutton, UK) [47], standardized by total count, and square-root transformed before the resemblance matrix was created. Similarity was determined by hierarchical agglomerative clustering of group means. A second stage NMDS of the samples was performed on a pairwise correlation matrix (Spearman Rank method) using the 2STAGE tool within Primer v6. This software was also used to identify the taxa primarily providing the variance between two decomposition stages of interest using the SIMPER tool.

## Results

### Soil chemistry

In the control plots without cadavers, soil chemistry did not significantly change over the course of the study. The pH of the control soils ranged from 6.34 to 6.58. Total extractable organic carbon and nitrogen ranged from 0.185 to 0.465 mg C per g dry weight soil (gdw^-1^) and 0.023–0.458 mg N gdw^-1^ (data not shown). There was also no significant change in ammonia, nitrate, and phosphate in the control soils with stage means ranging from 0.007 to 0.034 mg N gdw^-1^, 0.007 to 0.067 mg N gdw^-1^ and 0.067 to 0.224 ppm phosphate, respectively (data not shown).

In the soils below decaying cadavers, there were significant changes in soil chemistry. During Bloat-Active Decay there was a significant increase in extractable total organic carbon and total nitrogen, most of which was ammonia (Table 1). We also observed an increase in phosphate with three of the four cadavers (A3, C5 and D6); the fourth cadaver (B4) had considerably lower phosphate in both the control and CDI soils and this variability rendered the data non-significant. This increase in C and nutrients corresponded to the influx of decomposition fluids during Bloat-Active and remained significantly elevated in the cadaver decomposition islands (CDI) until the Advanced Decay III stage. Soil pH below the cadavers did not significantly increase nor decrease, and was not significantly different from the control soils, largely due to high variability between the four replicate cadavers.

**Table 1:** Soil pH and chemical concentrations in soils below decomposing cadavers.

	Initial	Bloat	Bloat- Active	Active	Active-Advanced	Advanced I	Advanced II	Advanced III
pH	6.678 ±0.151	6.030 ±1.156	7.049 ±1.214	6.595 ±0.964	6.797 ±0.906	6.876 ±0.929	7.192 ±0.897	6.470 ±0.946
PO_4_	0.186 ±0.162	0.160 ±0.189	0.283647 ±0.12	0.156 ±0.191	0.174 ±0.116	0.224 ±0.187	0.170 ±0.142	0.160 ±0.197
NO_3_ (mg N gdw^-1^)	0.021 ±0.017	0.170 ±0.273	0.118 ±0.169	0.070 ±0.095	0.027 ±0.061	0.036 ±0.046	0.172 ±0.221	0.045 ±0.054
NH_3_ (mg N gdw^-1^)	0.011^A^ ±0.008	0.084^A^ ±0.087	0.800^B^ ±0.687	1.502^BC^ ±0.924	2.253^BC^ ±0.726	3.846^C^ ±1.722	3.296^BC^ ±1.180	1.515^AB^ ±1.781
TN (mg N gdw^-1^)	0.046^A^ ±0.023	0.363^AB^ ±0.394	1.354^BC^ ±1.314	2.147^BC^ ±1.171	2.894^CD^ ±0.693	4.363^D^ ±1.416	3.979^CD^ ±1.294	2.078^ABC^ ±2.025
TOC (mg C gdw^-1^)	0.310^A^ ±0.245	0.637^AB^ ±0.417	1.427^ABC^ ±1.307	4.130^ABC^ ±2.594	4.814^ABC^ ±2.614	6.421^C^ ±1.969	5.866^BC^ ±3.165	4.920^ABC^ ±4.382

TN and TOC are total extractable nitrogen and organic carbon, respectively. Each number is the mean ± standard deviation of n = 4 cadavers. Statistically, stages that were significantly different from the others are indicated by different letter designation (p < 0.05, ranked ANOVA, Tukey-Kramer).

### Microbial activity

**Fig 1:**
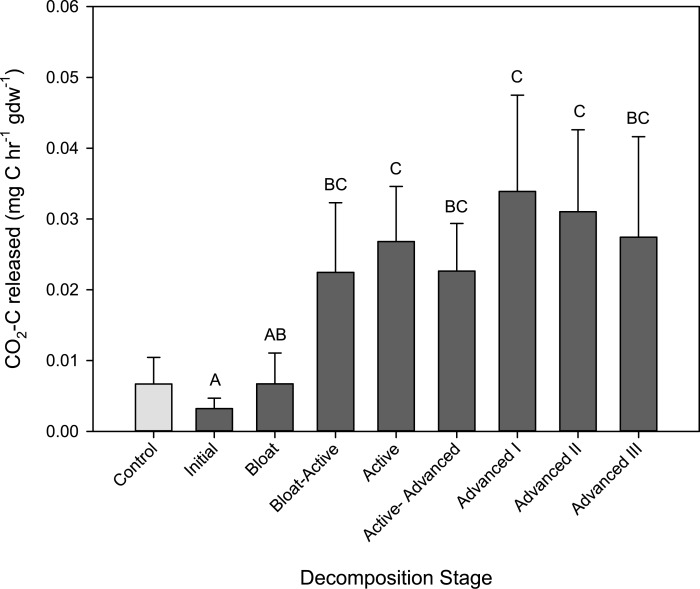
Microbial respiration rates in soils below decomposing cadavers. Means and standard deviations of n = 4 cadavers are presented for each stage; means with the same letter are not significantly different (p < 0.05, mixed model ranked ANOVA, Tukey-Kramer post-hoc). The light grey bar is the mean and standard deviation for control (no cadaver) soils throughout the duration of the study (n = 45).

**Fig 2:**
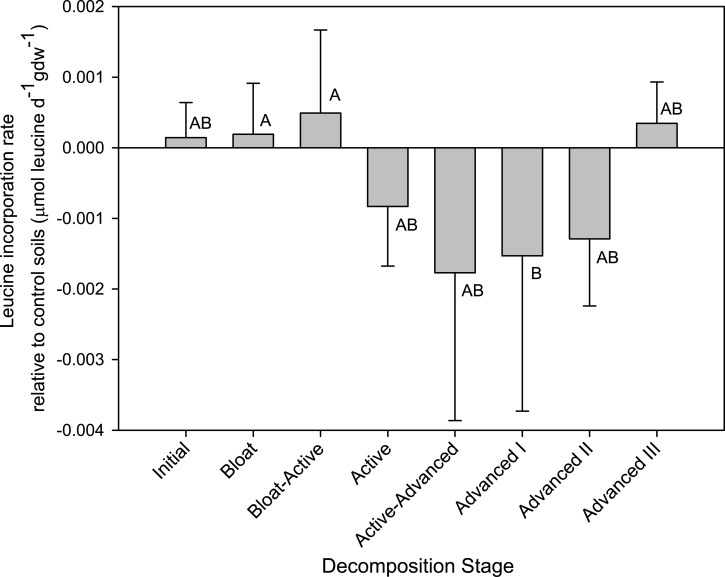
Microbial biomass production rates in soils below decomposing cadavers. Leucine incorporation rates expressed as mean difference between cadaver decomposition island (CDI) soils and their respective control soils (n = 4 cadavers); error bars are standard deviations. Means with the same letter are not significantly different (p < 0.05, mixed model ranked ANOVA, Tukey-Kramer post-hoc).

In the control soils, microbial respiration rate remained relatively constant over the course of the study with mean respiration rates of 0.004 to 0.009 mg C h^-1^ gdw^-1^ (data not shown). Mean biomass production rates varied from 0.000752 to 0.227 mmol ^3^H-leucine incorporated h^-1^ gdw^-1^. Because biomass production rate in control soils were quite variable, production rates in the soils below cadavers were normalized to rates in the control soils sampled on the same day. In soils below the cadavers, respiration rates and biomass production rates began to increase during the Bloat stage. The highest respiration rates were observed at two time points: The first local maximum was during Bloat-Active and Active Decay, the second maximum was during the Advanced I stage (Fig 1). The first increase in respiration during Active Decay was concomitant with the highest rates of biomass production (Fig 2), indicating a community with high microbial growth efficiency (i.e. high ratio of biomass production rates to respiration rates). Assuming a conversion factor of 0.54 kg C per mol ^3^H-leucine incorporated [48], these communities had a mean growth efficiency not significantly different from the control communities (0.57 ± 0.33%). Starting in Active Decay, biomass production rates in CDI soils declined to less than rates in control soils (Fig 2). Therefore the second period of maximum respiration rates observed during the Advanced Decay I stage corresponded with very low biomass production rates, and thus, low mean microbial growth efficiency (0.003 ± 0.001%). Respiration rates remained elevated through the final stages of decomposition, while biomass production rates returned to near starting values.

### Bacterial community structure and composition

**Fig 3:**
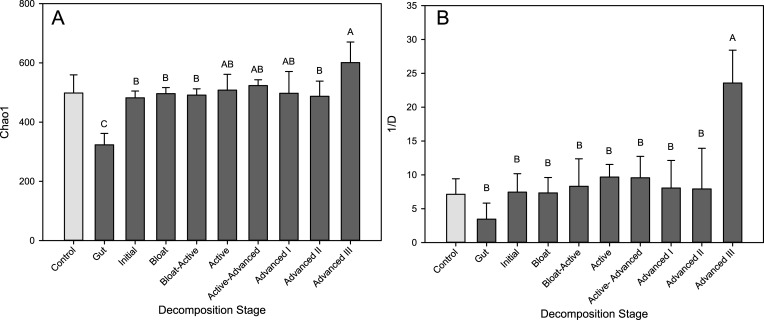
Richness and diversity of soil microbial communities below decomposing cadavers. A. Chao1 richness estimate, and B. Inverse of the Simpson diversity index, both calculated on libraries of equal size (121,340 sequences). Gut samples were also included for comparison. Mean and standard deviation of n = 4 cadavers are presented for each stage; means with the same letter are not significantly different (p < 0.05, mixed model ANOVA, Tukey-Kramer post-hoc). The light grey bar is the mean and standard deviation of the control (no cadaver) soil libraries throughout the duration of the study (n = 8).

Sequencing of amplified 16S rRNA genes resulted in 11,147,851 reads (2,634,911 unique) of sufficient quality after filtering, which had a mean read length of 291 bp. Libraries were subsampled to n = 121,340 reads each. These sequence reads clustered into 1050 operational taxonomic units (OTUs), with a mean of 421 OTUs per library. Rarefaction curves (S1 Fig) and a Good’s Coverage estimate of 0.994 (S2 Table) indicated sequence coverage was sufficient. The richness and diversity (estimated by Simpson’s Index) of the communities in the CDI soils did not change significantly until the final stage (Advanced III), when a significant increase in both richness and diversity was observed (Fig 3). The communities from the control soil samples did not undergo any significant changes in richness or diversity compared to each other or the initial pre-placement soil samples (Fig 3, light grey bars).

**Fig 4:**
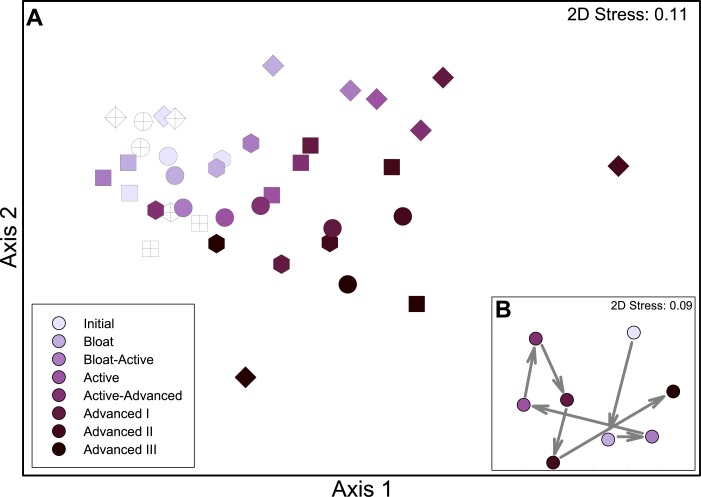
Microbial community structure in soils below decomposing cadavers. A. NMDS ordination of Bray Curtis dissimilarities between the relative abundance of bacterial OTUs. Shapes denote the 4 cadavers: A3 (square), B4 (diamond), C5 (hex), D6 (circle). Crossed symbols are control (no cadaver) samples. B. Second stage NMDS comparing the stages of decomposition, with arrows denoting order of stages through time.

Beta diversity of the microbial communities was analyzed using hierarchical agglomerative clustering of group mean Bray Curtis distances. As expected, the gut communities were compositionally quite different from the soil communities: gut microflora samples clustered together at 80.3% similarity and were only 65.4% similar to the soil samples (data not shown); CDI soil samples had 73.2% similarity with each other. NMDS visualization of Bray Curtis distances revealed shifts in CDI bacterial community composition as decomposition progressed (Fig 4A). For 3 of the 4 cadavers (A3, C5 and D6) the shifts during decomposition were similar (progressing from the upper left to lower right in Fig 4A). A shift in communities was also apparent the fourth cadaver (B4) (Fig 4A, diamonds) but the trajectory was slightly different from the other three, indicating that there may be some inter-individual variability in the succession of communities. A second stage clustering of decomposition stages for all four cadavers revealed that communities from the early stages (Bloat and Bloat-Active) are more similar to those taken prior to placement (Fig 4B). Then there was a noticeable change in community structure between the Bloat-Active and Active stages corresponding to when decomposition fluids enter the soil. Interestingly, the last stage (Advanced III) clusters with the early samples, suggesting a possible return toward the original community structure (Fig 4B).

**Fig 5:**
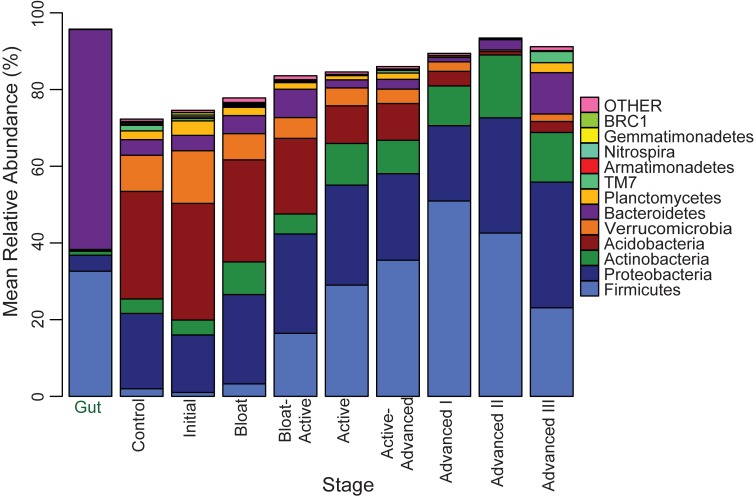
Bacterial phyla in soils below cadavers at different stages of decomposition. The data are the mean relative abundances of the 12 most abundance phyla from four cadavers.

**Fig 6:**
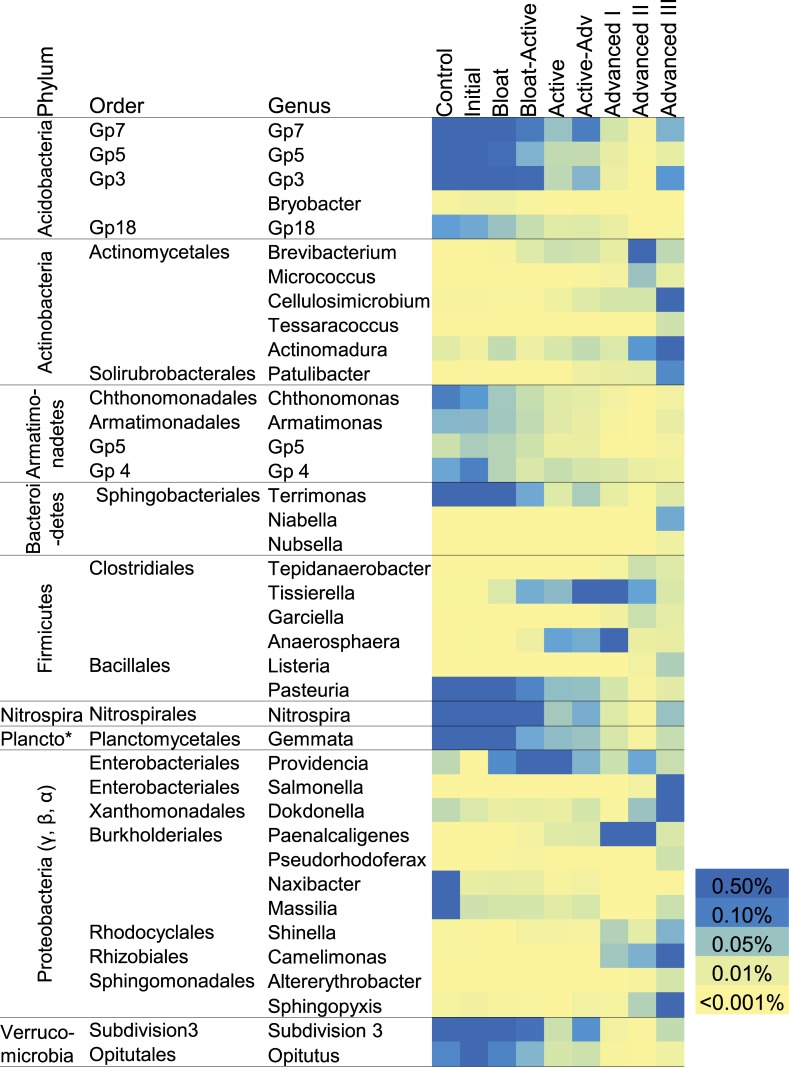
Bacterial OTUs that significantly change between stages of decomposition. Mean relative abundances (n = 4) of OTUs that are significantly different between stages (p < 0.05, ANOVA, Tukey-Kramer post-hoc). Only OTUs with more than 0.001% mean relative abundance in at least one stage and an effect size of 0.50 or larger are included here.

Unsurprisingly, the gut samples were dominated by Bacteroidetes (57%) and Firmicutes (32%) sequences, and showed little variability between the four cadavers. We observed a decrease in compositional variability in the soils below the four cadavers as decomposition progressed (Fig 5), indicating that the communities responded in a similar manner towards a common decomposer community. In these bacterial communities, the majority of the sequences assigned to Proteobacteria (ranging from 30 to 40% mean relative abundance for the 4 cadavers), Actinobacteria (15 to 23%) or Firmicutes (13 to 26%). Although the relative abundance of Proteobacteria as a phylum remained constant throughout the study, relative abundances of several Proteobacteria OTUs were significantly altered as decomposition progressed (Fig 6): Alphaproteobacteria and Gammaproteobacteria tended to increase (orders Enterobacteriales, Xanthomonadales, Rhizobiales, and Sphingomonadales), while Betaproteobacteria had a mixed response with some OTUs increasing (*Shinella*, *Paenalcaligenes*, *Pseudorhodoferax*) and others decreasing (*Naxibacter* and *Massilia*). The mean relative abundance of eleven other major phyla changed significantly between decomposition stages (Fig 5) (p < 0.05, ANOVA, Tukey-Kramer post hoc): Acidobacteria, Nitrospira, Verrucomicrobia and Armatimonadetes sequences were abundant before cadavers were introduced and during the early stages of cadaver decomposition, but declined to less than 15% of their original abundance during later stages. Within these phyla, several OTUs that were abundant in control and initial soils declined to < 0.1% in late Advanced Decay stages (Fig 6). Of note, several OTUs had slight increases in abundance in the final Advanced Decay III stage (Fig 6), likely contributing to the increased richness observed (Fig 3A), and return towards the original community composition (Fig 4B). Actinobacteria sequence abundances increased throughout decomposition, with the phyla increasing from 3.9% to 12.9%. Planctomycetes decreased during Bloat-Active through Advanced Decay II, but then returned to their original relative abundance in Advanced III. Firmicutes followed an opposite pattern to Planctomycetes, increasing during decay, with the phylum relative abundance reaching a maximum of 50.9% during Advanced I and declining to 23% by Advanced III. This was driven by several Firmicutes OTUs. An exception was *Pasteuria* spp. (Order Bacillialies), an obligate parasite of invertebrates, which was abundant initially and declined through decomposition. Phylum Bacteroidetes relative abundances peaked during Bloat-Active (7.4%) due in part to the proliferation of *Terrimonas*, and again in Advanced III (10.7%), due in part to proliferation of *Niabella* and *Nubsella*.

We specifically examined the OTUs which explained the greatest variability between successional communities using SIMPER analysis in Primer. In particular, we examined the difference between communities in Bloat-Active and Active (stage 3 and 4) which had the biggest compositional change (Fig 4B), as well as a comparison of communities from Active (stage 4) and Advanced I (stage 6) since these were the communities exhibiting the highest rates of respiration. In both cases, the analysis revealed that the differences in communities were due to changes in many taxa with relatively small contributions, the highest contribution being 0.36% (data not shown).

### Tracking human-associated bacteria in soil

**Fig 7:**
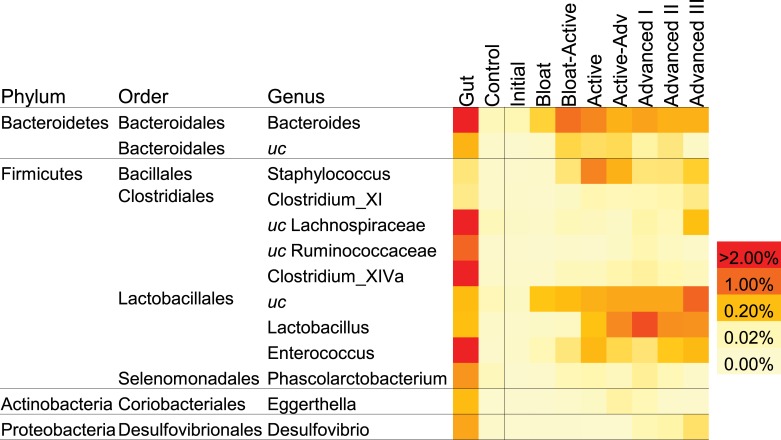
Human-associated bacterial OTUs detected in soils below decomposing cadavers. Mean relative abundances (n = 4) by decomposition stage. These OTUs were selected because they were present at > 0.05% in gut communities < 0.02% in the initial and control soils and > 0.02% in soils below cadavers during decomposition. *uc* = unclassifiable with at least 80% confidence using the RDP classifier.

**Fig 8:**
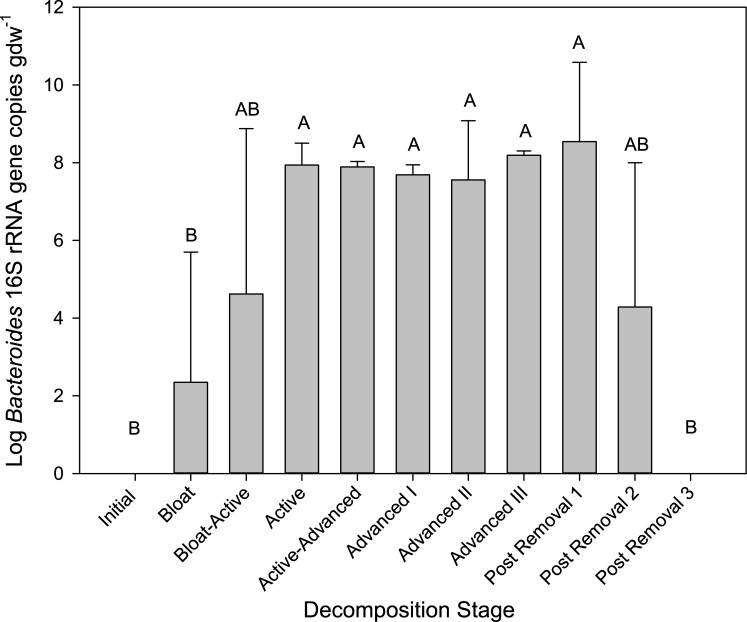
Abundance of human-associated *Bacteroides* in soils below decomposing cadavers. Mean and standard deviation (n = 3 cadavers) abundances for each decomposition stage; means with the same letter are not significantly different (p < 0.05, mixed model ANOVA, Tukey-Kramer post-hoc).

To test the hypothesis that human-associated microbes entered the soil, and to determine their persistence throughout decomposition, we examined the relative abundance of human-specific OTUs that appeared in the soils during decomposition (Fig 7). OTUs were selected that had relative abundances of > 0.05% in gut communities, < 0.02% in the initial and control soils, and > 0.02% in CDI soils. We found several that met these criteria: *Bacteroides*, *Staphylococcus*, and *Enterococcus* OTUs peaked in abundance during Active Decay. *Lactobacillus*, *Phascolarctobacterium*, and *Eggerthella* OTUs peaked during early Advanced Decay (Fig 7). In addition, we specifically quantified human-associated *Bacteroides*, one of the most common gut bacteria, using a highly sensitive qPCR assay targeting its 16S rRNA genes [45] for 3 of the 4 cadavers (C5 was not included in this analysis due to early removal of remains). During decomposition, there was a significant increase in human *Bacteroides* genes after Bloat-Active when decomposition fluids enter the soil (p < 0.05, Wilcoxon Rank Sum test) (Fig 8), with quantities remaining elevated through Advanced Decay III. Total bacterial 16S rRNA counts measured via qPCR in the CDI soils were not significantly different than their respective controls (data not shown, mixed model ranked ANOVA), so changes in *Bacteroides* abundances were not due to changes in total bacterial abundances. Because of the unexpected persistence of *Bacteroides*, we additionally examined their persistence in soils after the cadaver remnants were removed from the surface for 3 cadavers (removal occurred at 340, 303, and 219 days after placement for A3, B4 and D6 respectively). *Bacteroides* gene copies remained high 4 days after removal, decreased by 126 days, and were not detected at 204 days after removal.

## Discussion

Cadavers decomposing on the soil surface caused significant changes to the soils and soil microbial communities below. As would be expected upon the introduction of a complex mixture of substrates from cadaver decomposition products, there were changes in many taxa contributing to the succession of microbial community structure. We documented both functional and compositional shifts in microbial communities. In particular, the major changes seem to correspond to two distinct times in the decay process: first, when the body integrity was compromised and decomposition fluids first enter the soil (Bloat to Active Decay stages) and second, once most tissue was decomposed and mass loss rates of the cadaver slowed (Active to Advanced Decay stages).

Prior to placement of the cadavers, the initial (and control soil) bacterial communities were typical of a forest soil: Proteobacteria, Acidobacteria, Verrucomicrobia, Bacteroidetes, Actinobacteria and Planctomycetes dominated [49]. Once the body integrity was compromised (Bloat and Bloat-Active stages), a rapid flush of decomposition fluids entered the soil, increasing extractable carbon and nitrogen (particularly ammonia), and elevating respiration rates. As total bacterial abundances were not significantly different from control soils, this increase in activity was due to changes in metabolism and/or community structure. Indeed, biomass production rates were elevated, resulting in microbial growth efficiencies similar to control soils. In addition, the input of new N-rich substrates resulted in elevated N mineralization. This enhanced microbial N turnover has been documented during carcass decomposition [8] as would be expected for a N-limited forest soil community [50].

The Active Decay bacterial communities were different from the original soil community: this community was dominated by Proteobacteria and Firmicutes, with decreased relative abundances of Verrucomicrobia, Planctomycetes, and Acidobacteria compared to the initial communities. Verrucomicrobia and Planctomycetes generally have high metabolic requirements [51], they were likely outcompeted by fast-growing organisms (e.g. Proteobacteria) responding to the initial input of new, labile substrates. Acidobacteria abundances are often correlated to soil pH [52], however the pH ranges observed in our study (pH 5.5 to 7.5) were too small to explain the changes. Instead, their decline could be due to the fact that Acidobacteria often exhibit oligotrophic attributes: Fierer et al. [53] found this phylum most abundant in soils with low resource availability and least abundant in high organic carbon- amended soils. In addition to the selection of soil flora imposed by the newly introduced substrates, we also observed human-associated bacteria in the soils that were absent or rare in the initial soil communities. Interestingly, the relative abundance of Bacteroidetes, another common gut phylum, did not significantly change; this was due to a concurrent increase in human-associated genera (e.g. human-associated *Bacteroides*) and decrease in soil genera (e.g. *Terrimonas*). Metcalf et al. [23] observed similar changes in phyla in soils below decomposing mice in a controlled lab study: Firmicutes increased, Acidobacteria decreased, and Bacteroidetes did not change.

The second major transition occurred when decay rates begin to slow (Active-Advanced stage). Soil carbon and nutrients were still elevated, but there was a change in microbial activity. Respiration rates exhibited a second spike in early Advanced Decay, however unlike Active Decay, this was accompanied by a dramatic decrease in biomass production rates and ammonia accumulation. Since the leucine incorporation method has been shown to work equally well in oxic and anoxic environments [54], these low rates are indicative of a functionally distinct community compared to that of Active Decay: one with low growth efficiency and slow N mineralization. Again, total bacterial abundances had not changed significantly, so activity changes were due to changes in metabolic strategies and/or community composition.

Indeed, the microbial community structure during Advance Decay was different from that of the Active Decay communities. This bacterial community was dominated by Firmicutes, representing over half of the community. Within Firmicutes, we observed decreased abundances of aerobic Bacillales, and increased abundances of anaerobic Clostridiales (*Tissierella* and *Anaerosphaera*) and Lactobacillales (*Lactobacillus* and *Phascolarctobacterium*). We also observed increases in the anerobic *Paenalcaligenes* and *Eggerthella*. Lactobacillales and *Eggerthella* were present in the guts of these cadavers, indicating a likely human origin. *Tissierella* and *Anaerosphaera* have been identified in other anoxic environments (e.g. [55,56]). *Paenalcaligenes* spp. have been isolated from several environments, including *Hermetia illucens* (Black Soldier Fly) larvae [57], an insect frequently found on cadavers during late stages of decay [58], suggesting a possible entomological origin. Regardless of origin, the proliferation of these anaerobes and low microbial growth efficiency at this stage of decay indicates that oxygen had become limited in these soils and anaerobic metabolisms more prevalent.

Decomposition products have been shown to remain in soils for months after carcasses decay [21]. In our study, carbon and nutrients slowly declined as Advanced Decay progressed. This was accompanied by a decline in respiration rates. Biomass production rates began to recover. The anaerobic Firmicutes declined in the later stages and we observed blooms of a variety of taxa, including Actinobacteria, Alphaproteobacteria, Gammaproteobacteria, and Bacteroidetes, contributing to an increase in richness and diversity in the final stage. The late appearance of these taxa suggest they may have been using more chemically complex decomposition products, secondary metabolites, or recycling dead microbial cells. The clustering of the final stage communities with the beginning communities (Fig 4B), suggests that these communities may have been resilient to the perturbation (vis a vis [59]), however a longer time scale would be needed to determine if this was the case. Certainly the persistence of human-associated taxa indicates that even after > 83 days, the communities remain altered.

Decomposition is a high variable process, and it was noted that one of the four cadavers (B4) had a decomposition community structure distinct from the other three. The gut flora of this cadaver was similar to the others, and the initial and control soil communities from the plot clustered with the other plots, however the trajectory of the community during decay was slightly different. This could have been due to environmental differences: the plot was on a greater slope than the others and the cadaver was placed during an unusually hot time period in the summer, experiencing the highest mean temperature through active decay stage (27.8°C, compared with 22.4, 26, and 17.2°C for the other cadavers). Regardless of these inter-cadaver differences, the major trends in functional and structural succession described above held true for all four cadavers, indicating a common decay community progression.

We specifically hypothesized that human-associated microbes would enter the soil but not persist outside their host environment. As decomposition fluids entered the soil, we documented the appearance of several OTUs classified as known human commensals that were rare or non-existent in initial and control soils. Surprisingly, signatures of the obligate anaerobe *Bacteroides* persisted in high abundances in the CDI soils throughout the entire duration of the experiment, up to 198 days after cadaver placement. Once the dry remains of the cadavers were removed, abundances declined to below detection. While the presence of DNA sequences does not reveal if the organisms were viable, this observation does suggest that the reduced oxygen environment created by the tissue remnants was favorable for the anaerobic *Bacteroides*. The fate of human-associated microbes in the environment has been examined for some pathogens, e.g. *Salmonella* spp., *Camplyobacter* spp. and *Escherichia coli* O157 [60,61]. Survival rates varied depending on environmental conditions, but in all cases, the organisms declined in the soil environment, attributed largely to predation and competition from the indigenous soil community [62–65]. Due to the absence of comparable studies with *Bacteroides*, we can only speculate on the possible explanation for their persistence. It is likely that a combination of favorable conditions allowed them to persevere in the soil environment: pH ranges between five and eight, clayey soils with high water retention, anoxia, and an availability of SOM adsorption sites all increased survival times of fecal bacteria in soil [60,66]. Anoxic soils have been shown to share some species with vertebrate gut environments [37]. Now that we know these human-associated microflora persist much longer than expected, it raises questions about the interactions with indigenous soil microbes and their role in decomposition. The persistence of human-associated taxa in soil also suggests that they may be useful as biomarkers of human decomposition for forensic applications, since they are rare in natural soils and increase in abundance during the later stage of decay. The relationship of human microflora to time since death has been examined for bacterial communities on or inside human cadavers [25,26]; here we demonstrate that soil communities, too, exhibit postmortem patterns. Using soil communities as forensic evidence could prove useful in cases where body remains have been moved from the original location of decomposition.

## Conclusions

In summary, this study has revealed that microbial communities in soils below decomposing cadavers undergo distinct functional and structural changes. In particular, we noted that during Active Decay (period of most rapid mass loss), microbial growth efficiency was high and communities are dominated by more opportunistic, aerobic decomposers. When decomposition rates slow at the onset of Advanced Decay, anaerobic taxa proliferate and microbial growth efficiencies are low. We have additionally provided evidence that human-associated microbes persist in the soils for surprisingly long periods of time, suggesting a possible role in decomposition. Their low abundances in natural soils and significant increases during decay render them good candidates as biomarkers for long-term postmortem interval estimates.

## Supporting Information

S1 Fig Sample collection curves for 16S rRNA gene libraries from soil (black) and gut samples (grey). (TIF)

S1 Table Samples collected from four decaying cadavers (A3, B4, C5, D6) according to decomposition stage. The sample code is the cadaver code followed by the number of days after placement. For each, samples were collected from below the cadavers (cadaver decomposition island, CDI) and from a control site 2 m away with no cadaver. 16S rRNA amplicons sequencing was performed only on the bulleted samples. The entire data set was used for all other analyses.
(DOCX)

S2 Table 16S rRNA gene library statistics. (DOCX)

S1 Text Mothur code for sequence quality screening. (DOCX)
