# Supplementary figures and images for: Functional and Structural Succession of Soil Microbial Communities below Decomposing Human Cadavers
Source: PLoS One. 2015 Jun 12;10(6):e0130201. doi: 10.1371/journal.pone.0130201 (PMC4466320; doi:10.1371/journal.pone.0130201)

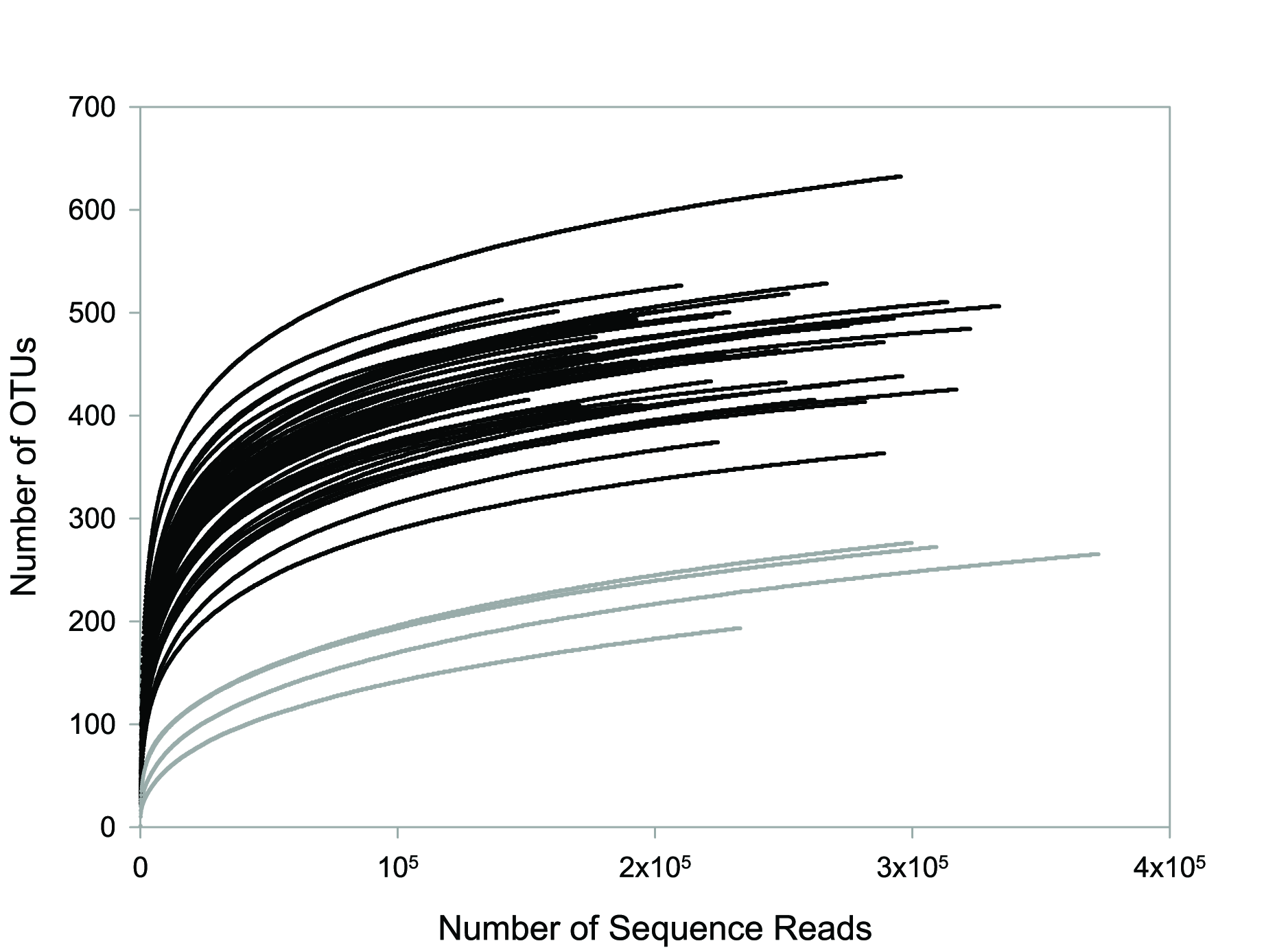

Supplement: S1 Fig — (TIF) [file pone.0130201.s001.tif]
