# Supplementary material for: Functional and Structural Succession of Soil Microbial Communities below Decomposing Human Cadavers
Source: PLoS One. 2015 Jun 12;10(6):e0130201. doi: 10.1371/journal.pone.0130201 (PMC4466320; doi:10.1371/journal.pone.0130201)
Supplement: S1 Table — The sample code is the cadaver code followed by the number of days after placement. For each, samples were collected from below the cadavers (cadaver decomposition island, CDI) and from a control site 2 m away with no cadaver. 16S rRNA amplicons sequencing was performed only on the bulleted samples. The entire data set was used for all other analyses. (DOCX) [file pone.0130201.s002.docx]

**S1 Table. Samples collected from four decaying cadavers (A3, B4, C5, D6) according to decomposition stage.**

|  | **A3** | | **B4** | | **C5** | | **D6** | |
| --- | --- | --- | --- | --- | --- | --- | --- | --- |
| **Stage** | **CDI** | **Control** | **CDI** | **Control** | **CDI** | **Control** | **CDI** | **Control** |
| Initial | A3-1• | A3-1C | B4-1• | B4-1C | C5-1• | C5-1C | D6-1• | D6-1C |
| Bloat | A3-2  A3-4• | A3-2C  A3-4C | B4-3  B4-4• | B4-3C  B4-4C | C5-4• | C5-4C | D6-4• | D6-4C |
| Bloat-Active | A3-6•  A3-8 | A3-6C  A3-8C | B4-5•  B4-6 | B4-5C  B4-6C | C5-5  C5-6• | C5-5C  C5-6C | D6-6• | D6-6C |
| Active | A3-10  A3-12• | A3-10C  A3-12C• | B4-7•  B4-8 | B4-7C•  B4-8C | C5-8• | C5-8C• | D6-8• | D6-8C• |
| Active-Advanced | A3-14  A3-16• | A3-14C  A3-16C | B4-9• | B4-9C | C5-10• | C5-10C | D6-10•  D6-13 | D6-10C  D6-13C |
| Advanced Decay I | A3-23• | A3-23C | B4-10  B4-13• | B4-10C  B4-13C | C5-12•  C5-16 | C5-12C  C5-16C | D6-17•  D6-23  D6-29 | D6-17C  D6-23C  D6-29C |
| Advanced Decay II | A3-46• | A3-46C | B4-25  B4-48• | B4-25C  B4-48C | C5-28  C5-39• | C5-28C  C5-39C• | D6-72• | D6-72C |
| Advanced Decay III | A3-87• | A3-87C• | B4-198• | B4-198C• | C5-83• | C5-83C | D6-114• | D6-114C• |

The sample code is the cadaver number followed by the number of days after placement. For each samples were collected from below the cadavers (cadaver decomposition island, CDI) and from a control site 2 m away with no cadaver. 16S rRNA amplicons sequencing was performed only on the bulleted samples. The entire data set was used for all other analyses.
