# Supplementary material for: Functional and Structural Succession of Soil Microbial Communities below Decomposing Human Cadavers
Source: PLoS One. 2015 Jun 12;10(6):e0130201. doi: 10.1371/journal.pone.0130201 (PMC4466320; doi:10.1371/journal.pone.0130201)
Supplement: S2 Table — (DOCX) [file pone.0130201.s003.docx]

**S2 Table. 16S rRNA gene library statistics.**

| Cadaver | Sample type | Sample name | Number of raw reads | Number of reads post-quality filtering | Sub-sampled size | Good's Coverage | Number of OTUs | Inverse Simpson's Index |
| --- | --- | --- | --- | --- | --- | --- | --- | --- |
| A3 | Gut | A3-F | 442191 | 320842 | 121340 | 0.9995 | 193 | 6.92 |
| A3 | Soil | A3-1 | 275409 | 186113 | 121340 | 0.9995 | 466 | 5.27 |
| A3 | Soil | A3-4 | 298034 | 205019 | 121340 | 0.9994 | 445 | 4.59 |
| A3 | Soil | A3-6 | 265479 | 181621 | 121340 | 0.9994 | 457 | 6.48 |
| A3 | Soil | A3-12 | 278874 | 201388 | 121340 | 0.9994 | 481 | 9.34 |
| A3 | Control soil | A3-12C | 311724 | 220164 | 121340 | 0.9994 | 514 | 9.51 |
| A3 | Soil | A3-16 | 323117 | 237528 | 121340 | 0.9994 | 453 | 5.92 |
| A3 | Soil | A3-23 | 337683 | 230732 | 121340 | 0.9994 | 420 | 4.19 |
| A3 | Soil | A3-46 | 386446 | 288581 | 121340 | 0.9995 | 425 | 3.36 |
| A3 | Soil | A3-46R | 260550 | 189060 | 121340 | 0.9994 | 382 | 5.32 |
| A3 | Soil | A3-87 | 419890 | 327947 | 121340 | 0.9993 | 462 | 18.85 |
| A3 | Control soil | A3-87C | 308530 | 211247 | 121340 | 0.9994 | 490 | 7.51 |
| B4 | Gut | B4-F | 488777 | 386484 | 121340 | 0.9993 | 194 | 2.17 |
| B4 | Soil | B4-1 | 257181 | 157702 | 121340 | 0.9994 | 407 | 6.34 |
| B4 | Soil | B4-4 | 288657 | 199856 | 121340 | 0.9994 | 398 | 9.61 |
| B4 | Soil | B4-5 | 358151 | 271151 | 121340 | 0.9994 | 407 | 4.77 |
| B4 | Soil | B4-7 | 417950 | 329738 | 121340 | 0.9994 | 400 | 8.01 |
| B4 | Soil | B4-7R | 384435 | 296223 | 121340 | 0.9994 | 396 | 9.34 |
| B4 | Control soil | B4-7C | 283427 | 200094 | 121340 | 0.9995 | 401 | 5.43 |
| B4 | Soil | B4-9 | 394683 | 308424 | 121340 | 0.9993 | 416 | 8.69 |
| B4 | Soil | B4-13 | 325913 | 234827 | 121340 | 0.9994 | 355 | 5.93 |
| B4 | Soil | B4-48 | 413897 | 298956 | 121340 | 0.9993 | 340 | 8.97 |
| B4 | Soil | B4-198 | 407648 | 306786 | 121340 | 0.9991 | 593 | 20.53 |
| B4 | Control soil | B4-198C | 273142 | 182658 | 121340 | 0.9995 | 395 | 4.98 |
| C5 | Gut | C5-F | 331892 | 237054 | 121340 | 0.9993 | 150 | 1.72 |
| C5 | Soil | C5-1 | 267540 | 176062 | 121340 | 0.9994 | 433 | 6.76 |
| C5 | Soil | C5-4 | 313442 | 202062 | 121340 | 0.9994 | 445 | 8.75 |
| C5 | Soil | C5-6 | 347282 | 260497 | 121340 | 0.9994 | 423 | 14.10 |
| *C5 | Soil | C5-8 | 23135 | 13770 | N/A | N/A | N/A | N/A |
| C5 | Soil | C5-8C | 398169 | 304757 | 121340 | 0.9993 | 482 | 13.16 |
| C5 | Soil | C5-10 | 251183 | 169928 | 121340 | 0.9995 | 493 | 10.14 |
| C5 | Soil | C5-12 | 363480 | 278872 | 121340 | 0.9994 | 519 | 13.55 |
| C5 | Soil | C5-39 | 420874 | 334725 | 121340 | 0.9995 | 470 | 3.35 |
| C5 | Soil | C5-39R | 391175 | 299827 | 121340 | 0.9995 | 459 | 3.22 |
| C5 | Control soil | C5-39C | 342581 | 240597 | 121340 | 0.9994 | 488 | 6.13 |
| C5 | Soil | C5-83 | 205667 | 145904 | 121340 | 0.9994 | 506 | 29.59 |
| D6 | Gut | D6-F | 421781 | 319974 | 121340 | 0.9994 | 204 | 3.02 |
| D6 | Soil | D6-1 | 185787 | 126737 | 121340 | 0.9995 | 435 | 11.43 |
| D6 | Soil | D6-4 | 298905 | 187166 | 121340 | 0.9994 | 438 | 6.40 |
| D6 | Soil | D6-6 | 335492 | 231901 | 121340 | 0.9995 | 480 | 7.91 |
| D6 | Soil | D6-8 | 342346 | 263942 | 121340 | 0.9995 | 483 | 11.69 |
| D6 | Control soil | D6-8C | 256704 | 179271 | 121340 | 0.9995 | 403 | 4.72 |
| D6 | Soil | D6-10 | 193849 | 149615 | 121340 | 0.9995 | 472 | 13.53 |
| D6 | Soil | D6-17 | 364754 | 287668 | 121340 | 0.9994 | 477 | 8.54 |
| D6 | Soil | D6-17R | 435694 | 350524 | 121340 | 0.9994 | 496 | 7.14 |
| D6 | Soil | D6-72 | 333389 | 260324 | 121340 | 0.9993 | 438 | 16.00 |
| D6 | Soil | D6-114 | 340608 | 263285 | 121340 | 0.9993 | 493 | 25.32 |
| D6 | Control soil | D6-114C | 261797 | 177722 | 121340 | 0.9995 | 437 | 5.68 |

R indicates replicate library from the same soil sample (technical replicate). *Sample was removed from analyses due to insufficient sequence coverage.
