# Supplementary material for: Functional and Structural Succession of Soil Microbial Communities below Decomposing Human Cadavers
Source: PLoS One. 2015 Jun 12;10(6):e0130201. doi: 10.1371/journal.pone.0130201 (PMC4466320; doi:10.1371/journal.pone.0130201)
Supplement: S1 Text — (DOCX) [file pone.0130201.s004.docx]

**S1 Text. Mothur code for sequence quality screening.**

#ARF2012 16S libraries

#Mothur v.1.33.3

#MiSeq, 16S rRNA, V4, 150bp paired end reads, primers 515F and 806R (Caporaso 2012)

#made a "arf.files" file containing sample name, forward and reverse fastq file name.

#prepared alignment file: trim reference alignment to V4 and rename

pcr.seqs(fasta=silva.bacteria/silva.bacteria.fasta, keepdots=F, start=11894, end=25319)

#renamed silva.bacteria.v4.fasta; nbases 270-351

#join forward and reverse reads

make.contigs(file=arf.files, processors=8)

summary.seqs(fasta=arf.trim.contigs.fasta, processors=8)

##15,948,986 total, length 148-303, ambig 0-131, polymer 2-151

screen.seqs(fasta=arf.trim.contigs.fasta, group=arf.contigs.groups, optimize=none, start=1, maxambig=0, maxhomop=8, minlength=290, maxlength=295, processors=8)

summary.seqs(fasta=arf.trim.contigs.good.fasta)

##11,667,539 total

unique.seqs(fasta=arf.trim.contigs.good.fasta)

summary.seqs(count=current, processors=8)

##11,667,539 total; 4,449,236 unique

count.seqs(group=arf.contigs.good.groups, name=arf.trim.contigs.good.names, processors=8)

align.seqs(fasta=arf.trim.contigs.good.unique.fasta, reference=silva.bacteria.v4.fasta, flip=T, processors=8)

summary.seqs(count=arf.trim.contigs.good.count_table, fasta=arf.trim.contigs.good.unique.align)

##11,667,539 total; 4,449,236 unique

screen.seqs(count=arf.trim.contigs.good.count_table, fasta=arf.trim.contigs.good.unique.align, summary=arf.trim.contigs.good.unique.summary, optimize=none, start=1, end=13424)

summary.seqs(count=arf.trim.contigs.good.good.count_table, fasta=arf.trim.contigs.good.unique.good.align)

##11,599,700 total; 4,380,900 unique, length 1-13424

filter.seqs(fasta=arf.trim.contigs.good.unique.good.align, vertical=T, trump=.)

summary.seqs(count=current, fasta=arf.trim.contigs.good.unique.good.filter.fasta)

##11,599,700 total; 4,380,900 unique; length 1-664

pre.cluster(count=arf.trim.contigs.good.unique.good.filter.count_table, fasta=arf.trim.contigs.good.unique.good.filter.fasta, diffs=2, processors=8)

summary.seqs(count=arf.trim.contigs.good.unique.good.filter.precluster.count_table, fasta=arf.trim.contigs.good.unique.good.filter.precluster.fasta)

##11,599,700 total; 2,814,868 unique; length 1-664

chimera.uchime(count=arf.trim.contigs.good.unique.good.filter.precluster.count_table, fasta=arf.trim.contigs.good.unique.good.filter.precluster.fasta, reference=self, dereplicate=T, processors=8)

remove.seqs(accnos=arf.trim.contigs.good.unique.good.filter.precluster.uchime.accnos, count=arf.trim.contigs.good.unique.good.filter.precluster.uchime.pick.count_table, fasta=arf.tim.contigs.good.uniuqe.good.filter.precluster.fasta)

##removed 165,304 sequences

summary.seqs(count=arf.trim.contigs.good.uniuqe.good.filter.precluster.uchime.pick.pick.count_table, fasta=arf.trim.contigs.good.uniuqe.good.filter.precluster.pick.fasta)

##11,222,945 total; 2,649,564 unique

classify.seqs(count=arf.trim.contigs.good.uniuqe.good.filter.precluster.uchime.pick.pick.count_table, fasta=arf.trim.contigs.good.uniuqe.good.filter.precluster.pick.fasta, reference=trainset9_032012.pds.fasta, taxonomy=trainset9_032012.pds.tax, cutoff=80)

remove.lineage(count=current, fasta=current, taxonomy=arf.trim.contigs.good.unique.good.filter.precluster.pick.pds.wang.taxonomy, taxon=Chloroplast-Mitochondria-unknown-Eukaryota-Archaea)

summary.seqs(count=arf.trim.contigs.good.unique.good.filter.precluster.uchime.pick.pick.pick.count_table, fasta=arf.trim.contigs.good.uniuqe.good.filter.precluster.pick.pick.fasta)

##11,174,851 total; 2,634,911 unique

##renamed files to arf.taxonomy, arf.fasta, arf.count_table, arf.groups (from arf.contigs.good.groups) and arf.names(from arf.trim.conitgs.good.names)

##files for MG-RAST upload

degap.seqs(fasta=arf.fasta)

split.groups(fasta=arf.ng.fasta, count=arf.count_table)

phylotype(taxonomy=arf.taxonomy)

#output arf.tx.sabund, arf.tx.rabund, arf.tx.list

make.shared(list=arf.tx.list, count=arf.count_table, label=1)

classify.otu(list=arf.tx.list, count=arf.count_table, taxonomy=arf.taxonomy, label=1)

#output: arf.tx.1.cons.taxonomy, arf.tx.1.cons.tax.summary

count.groups(shared=arf.tx.shared)

##minimum 121,340 (this will drop one sample, 50-8, which has an usually low 13,324 reads)

sub.sample(shared=arf.tx.shared, size=121340)

#output: arf.tx.1.subsample.shared

##alpha diversity

summary.single(shared=arf.tx.1.subsample.shared, calc=nseqs-coverage-sobs-chao-invsimpson)
